# An Essay on Child-Bed Fever, Read before the Medical Association of the State of Georgia

**Published:** 1859-08

**Authors:** S. H. Dean

**Affiliations:** Conyers, Georgia


### ARTICLE II.

*An Essay on Child-Bed Fever, read before the Medical Association of the State of Georgia.*—By S. H. DEAN, M. D., of Conyers, Georgia.

The true nature and pathology of this disease was, for a long time, unknown. It was called a fever, regarded as a fever and treated as a fever; and doubtless many of its victims perished from the want of a proper knowledge of its pathology.

Dr. Gordon, of Abberdeen, Eng., learned from post-mortum dissections that the disease was not a fever; but an acute inflammation of the uterus and its appendages and of the peritoneum. That was an era in its history, and from that time, it has been treated upon rational principles, and with better success.

It is usually epidemic. Densely crowded and badly ventilated portions of large cities and hospitals seem to be its favorite abode; yet, it occasionally transcends its usual bounds, enters the circle where beauty and intelligence, refinement and affluance reign—it enters the elegant mansion in the most salubrious place; lays hold of its victim and often refuses to relinquish its grasp, until life is extinct.

CAUSE.—The cause of no disease is more obscure than that of Child-Bed Fever.

Mechanical injuries, exposure, contagion and miasmata are the chief causes to which it is ascribed.

That it does not arise from mechanical injuries is rendered evident, from the fact that women who have the easiest labor are just as liable to it, as those who have the most difficult.

Exposure or anything else that has a depressing effect upon the nervous system, may act as an exciting cause, but is insufficient to produce the disease.

Contagion has been regarded as the cause, by many of our best writers upon the subject; and were we to look, only, to the evidence in its favor, it would seem to be overwhelming.

If it is contagious, how can an accoucheur communicate it to one-third of his patients, while two-thirds may escape ?

If contageous, why does its epidemics always make their appearance first, in crowded hospitals, and in the filthy and badly ventilated portions of large cities where the poor and destitute are congregated ?

Contagious diseases, usually, require an incubation of from a few days to several weeks, but this disease may be produced in a day.

MIASM.—Since this disease almost always makes its appearance first, in localities fruitful of miasmatic or endemic diseases, it is reasonable to attribute it to the same or to analagous causes.

We know nothing of the properties of this hypothetical miasm or influence. Like malaria, it is not susceptible to the most delicate test of the chemist, nor to the most searching scrutiny of the microscopist. It may be effluvial or telluric, but we know not from "whence it cometh or whither it goeth."

The experiments of Dr. Collins, of Dublin, and Van Busch, of Berlin, are the strongest arguments in support of the endemic or miasmatic theory.

Dr. Collins having charge of the Dublin Lying-in Hospital, in which the disease was prevaling, had the wards cleared, the windows closed and rooms filled with chlorine gas for forty-eight hours; after which the parts constructed of wood, were washed with chloride of lime, and the beds subjected to a temperature of from 120° to 130° F., and from that time the disease disappeared.

Child-bed fever was prevailing, virulently, in the Lying-in Hospital of the University of Berlin, in the early part of 1851. Dr. Bon Busch had the Hospital closed, and by the use of stoves elevated the temperature of the wards, to from 145° to 167° F., for 48 hours; and from that time there was not another case in that institution during that season, notwithstanding it continued to prevail in the city.

In the latter part of the same year a few other cases occurred, when the wards were heated as before, and from that time the disease was cut short.

Symptoms.—Child-bed fever, usually, manifests itself about the third day after parturition, but may do so sooner or may not before the sixth or seventh day.

A chill, usually, well marked, but sometimes only amounting to a slight chilliness, is the first symptom.

The chill is followed by a reaction.

The skin now becomes hot and dry; pulse frequent, small and wiry, but sometimes full and bounding.

The secretions are changed. The tongue is usually moist, but sometimes dry, especially, in the centre. Urine small in quantity, highly colored and voided with pain or difficulty. Mamma flaccid, lochia suppressed or diminished in quantity, and of a fetid odor. The bowels are constipated and distended with gas. Constant acute pain in the pelvic, iliac and hypogastric regions, which is greatly increased by pressure, coughing, &c.

The countenance is anxious, respiration hurried, and the patient lies in the dorsal position, with the lower extremities slightly flexed, so as to relax the abdominal, psoas and iliac muscles.

Cranialgia, insomnia, nausea and vomiting are often present.

The symptoms become more and more grave, as the disease advances.

The abdomen becomes more and more tympanitic, and so sensitive that the least pressure cannot be borne.

The pulse becomes very feeble and frequent, respiration excessively hurried; the strength fails, mental aberrations supervene and the patient sinks.

In cases of child-bed fever, where the patient is seen early, and promptly and properly treated, the disease often yields submissively. And when this is the case, the pain subsides, the pulse and respiration becomes more natural, the secretions return and the patient is soon convalescent.

If the peritoneum should not be involved in the inflammation, the symptoms will not be so well marked. There will be less pain, less nervous shock, less tympanitis and more natural respiration.

When metritis alone exists, the pain is of a dull aching character and may be mistaken for after pains or rheumatism of the uterus. To aid the diagnosis in such cases, the patient should be instructed to flex the thighs upon the pelvis, so as to cause the psoas and iliac muscles to compress the uterus; and if that should cause much pain, there may be reason to suspect the presence of inflammation. The foregoing symptoms are descriptive of the disease as it occurs in epidemics. Sporadic cases, in the country and small towns, are much milder and more easily controlled than cases which occur during an epidemic.

TREATMENT.—In the treatment of this disease, the physician should not allow himself to be misled, by the name "fever;" but should bear in mind, that he has to combat an acute inflammation of the uterus and its appendages and the peritoneum, which often require the use of the strongest antiphlogistic remedies.

Blood-letting is the sheet-anchor in all cases of acute sthenic inflammation, and is strongly indicated and remarkably well borne when the serous tissues are involved.

What physician has not seen the acute pain of pleuritis relieved while the blood was still flowing in a full stream? Just as it relieves pleuritis, so will it relieve metro-peritonitis, if resorted to sufficiently early and boldly.

Dr. Gordon, in speaking of the treatment of this disease, says, "When I had resolution to take twenty or twenty-four ounces at one bleeding, I disregarded it (the prejudice against bleeding) because I was sure that that quantity, taken away in six or eight hours after the attack, would certainly cure the disease." Again he says, "When I took away only ten or twelve ounces of blood from my patient, she always died; but when I had courage to take away twenty or twenty-four ounces at one bleeding in the beginning of the disease, the patient never failed to recover."—In order that this great remedy may be effectual, it should be resorted to early, and from sixteen to twenty four ounces of blood taken at once; unless it should be forbidden by prostration or syncope of the patient. When prostration supervenes, or the inflammation produces effusion, the lancet ceases to be a "saver of life unto life." It can then, only, increase the prostration and sink the feeble remains of life. Under such circumstances, instead of depleting remedies, supporting treatment is indicated.

MEDICATION.—Immediately after venesection, a brisk cathartic should be administered, and the patient brought speedily under the influence of veratrum viride. Veratrum is a powerful sedative, and controls the action of the heart, in inflammatory diseases, better than any other drug in the whole materia medica. Five drops (Norwood's Tincture,) may be given at a dose, and repeated every two, three or four hours, (according to the urgency of the symptoms, and the effect produced,) until the pulse is reduced to 70 or 80.* The system should be kept under its influence, until the inflammatory action subsides, or, until it ceases to control the pulse, which it is very apt to do, when prostration comes on.

The use of purgatives may be objected to, from the fact, that they increase the pain, by throwing the bowels into peristaltic action. This evil is much more than counterbalanced by the removal of gas and feces. When peritonitis exists, they deplete directly from the inflamed tissue, relieve congestion, prevent effusion, and perhaps, save the inflamed parts from destructive suppuration.

Many physicians have their favorite cathartics. None can be better than calomel, or calomel with rhubarb or comp. ext. of colocynth. It is reliable—does not produce griping—is not offensive to the stomach, but in large doses, acts as a sedative to it. 12 or 15 grains of calomel, with an equal amount of comp. ext. of colocynth, may be given immediately after venesection. If it should not produce free action upon the bowels in six hours, it may be followed by a teaspoonful of spt. turpentine mingled with two or

*If necessary, the size of the dose may be increased one drop each dose, until it amounts to 8 or 10 drops.

three tablespoonsful of castor oil. The spts. turpentine has a happy effect, causing the discharge of gas, which affords great relief to the patient.

After the bowels have been freely opened, a $\frac{1}{4}$ gr. dose of morphine may be given, and repeated at such intervals as may be necessary, in order to relieve pain, and procure that refreshment which sleep alone can give. Calomel, in small doses, may be given with the morphine, for its alterative effects, and to prevent costiveness. The morphine should not be used so freely as to prevent the action of the purgative, as it may be necessary, to have the bowels freely opened every day. If the pain should not be sufficiently relieved by the venesection, or, if it should recur, the venesection may be repeated.

Emolient poultices, or flannel dipped in hot water and vinegar, should be applied to the abdomen, to aid in relieving the pain. If these applications should fail to afford any relief, a large blister may be applied.

If the abdomen should be very tympanitic, some relief may be afforded by the use of the following prescription:

| | |
|---|---|
| ℞.—Magnesiæ carbonatis, | ʒiv, |
| Olei anise, | f ʒi, |
| Mannæ, | ℥i, |
| Aquæ, | f ℥vii. |
| M. | |

Take two tablespoonsful every hour or two, until it acts freely upon the bowels. Mucilaginous washes and anodyne injections, per vaginum, will often be of service.

If prostration should supervene, the plan of treatment must be changed.

All remedies which have a tendency to weaken the vital powers should be discontinued, and a supporting plan of treatment substituted. Wine whey, brandy punch, and beef tea will then be proper remedies.

Mild sporadic cases of puerperal fever, do not require so vigorous treatment as is recommended in the foregoing pages. In some of these cases, bleeding and blistering are not to be required; even the milder remedies need not to be used with a great deal of vigor.

